# Dissolution Dominating Calcification Process in Polar Pteropods Close to the Point of Aragonite Undersaturation

**DOI:** 10.1371/journal.pone.0109183

**Published:** 2014-10-06

**Authors:** Nina Bednaršek, Geraint A. Tarling, Dorothee C. E. Bakker, Sophie Fielding, Richard A. Feely

**Affiliations:** 1 NOAA Pacific Marine Environmental Laboratory, Seattle, Washington, United States of America; 2 British Antarctic Survey, High Cross, Cambridge, United Kingdom; 3 Centre for Ocean and Atmospheric Sciences, School of Environmental Sciences, University of East Anglia, Norwich Research Park, Norwich, United Kingdom; University of Western Sydney, Australia

## Abstract

Thecosome pteropods are abundant upper-ocean zooplankton that build aragonite shells. Ocean acidification results in the lowering of aragonite saturation levels in the surface layers, and several incubation studies have shown that rates of calcification in these organisms decrease as a result. This study provides a weight-specific net calcification rate function for thecosome pteropods that includes both rates of dissolution and calcification over a range of plausible future aragonite saturation states (Ω_ar_). We measured gross dissolution in the pteropod *Limacina helicina antarctica* in the Scotia Sea (Southern Ocean) by incubating living specimens across a range of aragonite saturation states for a maximum of 14 days. Specimens started dissolving almost immediately upon exposure to undersaturated conditions (Ω_ar_∼0.8), losing 1.4% of shell mass per day. The observed rate of gross dissolution was different from that predicted by rate law kinetics of aragonite dissolution, in being higher at Ω_ar_ levels slightly above 1 and lower at Ω_ar_ levels of between 1 and 0.8. This indicates that shell mass is affected by even transitional levels of saturation, but there is, nevertheless, some partial means of protection for shells when in undersaturated conditions. A function for gross dissolution against Ω_ar_ derived from the present observations was compared to a function for gross calcification derived by a different study, and showed that dissolution became the dominating process even at Ω_ar_ levels close to 1, with net shell growth ceasing at an Ω_ar_ of 1.03. Gross dissolution increasingly dominated net change in shell mass as saturation levels decreased below 1. As well as influencing their viability, such dissolution of pteropod shells in the surface layers will result in slower sinking velocities and decreased carbon and carbonate fluxes to the deep ocean.

## Introduction

Formation and dissolution of calcium carbonate (CaCO_3_), and carbon export from the surface to the deep ocean are important mechanisms in the global carbon cycle, immediately related to the control of atmospheric CO_2_ (carbon dioxide) and regulation of the dissolved CO_2_ concentration and pH [1]–[4]. CaCO_3_ can occur in calcite, aragonite, or high-magnesium calcite form, and different planktonic species produce shells or skeletons of one of these mineral forms. Aragonite is the more soluble form of CaCO_3_, and its formation and dissolution is determined by the CaCO_3_ saturation state (Ω_ar_), which is the product of the concentrations of calcium (Ca^2+^) and carbonate ions (CO_3_^2−^) at the in situ temperature, salinity, and pressure, divided by the apparent stoichiometric solubility product for the structural form of CaCO_3_ (K*_sp_):
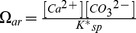
(1)


Surface waters are generally supersaturated with CO_3_^2−^ but their concentration tends to decrease with depth. Below the saturation horizon (Ω_ar_<1), the concentration of these ions decreases to the point where aragonite starts to dissolve [5]–[9]. Carbonate ions in the surface ocean buffer the increased uptake of atmospheric CO_2_, leading to a decrease in their concentration and a shallowing of the saturation horizon [2], [10]–[11]; this process is referred to as ocean acidification. The greater solubility of aragonite in colder waters means that ocean acidification effects will be most evident in polar regions [11]–[13]. For instance, the Southern Ocean is predicted to reach surface undersaturation seasonally by about 2038 [14].

Among the most vulnerable organisms to ocean acidification are pteropods, which are thin-shelled aragonite producers particularly abundant at high latitudes. Pteropods are a major component of polar ocean food webs, and they play a key role in energy transfer and carbon fluxes in these regions by exerting a high grazing pressure with large feeding webs, faeces, and pseudofaeces sinking rapidly and transferring carbon to the ocean interior [15]. Furthermore, as a ballast for other particulate organic matter [16], the rapid sinking and dissolution of the pteropod shells at depth is an important contributor of carbon and alkalinity in the deep ocean [5].

As pteropods exert little control over the pH and carbonate chemistry of their calcifying fluid, they are more sensitive to the effects of ocean acidification than other calcifying marine organisms [17]. Corals, for example, can elevate the pH of their calcifying fluid relative to the surrounding sea water, buffering ocean acidification effects [18]–[19]. Nevertheless, pteropods could potentially counteract the loss of shell through calcification processes within the shell interior, as occurs in aragonite-based corals [20]. Lischka et al. [21], for example, presented evidence of “repair” calcification in pteropods, with shell thickening at sites of previous shell dissolution.

The response of thecosome pteropods to aragonite saturation state in terms of rates of calcification was considered by Comeau et al. [22], who carried out incubations with the Arctic pteropod, *Limacina helicina*. They noted that calcification did not cease at Ω_ar_ levels below 1, but in fact was still evident at Ω_ar_ of 0.6. However, the rate of calcification was sensitive to saturation state, and demonstrated a logarithmic decrease from Ω_ar_ levels of 2.0 to 0.6 such that, by Ω_ar_ equal to 1, the calcification rate was less than half of that observed at Ω_ar_ of 2.0. The study only determined gross calcification rates but did not also assess whether such rates would be sufficient to counteract dissolution.

One approach to accounting for rates of dissolution is to apply dissolution kinetic algorithms as follows:
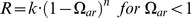
(2)where R is the rate of dissolution (%); k, the dissolution rate constant (d^−1^); and n, the dimensionless reaction order [6].

The dissolution rate constant for aragonite has been principally derived through studies on non-living biogenic material and used to estimate dissolution rates as part of the global aragonite cycle [6], [23]. Gangstø et al. [23] considered the dissolution of abiogenic aragonite to be a first order reaction (n = 1) with a dissolution rate constant (k) of 10.9 d^−1^. Such an approach is supported by observations of Bednaršek et al. [24], who showed that, in the natural environment as well as in laboratory experiments, dissolution of the shell of the Southern Ocean pteropod *L. helicina* can be rapid and substantial when exposed to Ω_ar_ levels near or below 1.0. Nevertheless, the rate of dissolution in living pteropods may not simply be a function of abiogenic dissolution kinetics since living individuals have specific shell structures and mechanisms that can slow down damage from dissolution processes [25]. Thus, it is important to verify that dissolution rates of abiogenic material agree with dissolution rates of living organisms.

In this study, we consider the consequences to the shells of living specimens of *Limacina helicina ant.* of exposure to seawater undersaturated for aragonite. We examine the shells in two ways to derive an overall level of gross dissolution: firstly, by examining the shell aperture to determine the penetration thickness of dissolution and/or any calcification, and secondly, by estimating the proportion of the surface shell undergoing dissolution. As the dissolution rate of aragonite is an important biogeochemical parameter, this study will determine whether it can be equally applied to living aragonite producers as it can to abiogenic aragonite. Finally, pteropods are a major source of CaCO_3_ to the deeper layers of the Southern Ocean, and any processes acting to decrease shell weight through dissolution will impact this flux. This study will examine how measured dissolution levels will impact the amount of pteropod-derived CaCO_3_ flux leaving the surface layers for eventual export to the ocean interior.

## Materials and Methods

### Sampling methods

Sampling and incubation were carried out aboard the RRS *James Clark Ross* during cruise JR177 to the Scotia Sea in January and February 2008 along a transect from 62.21°S 44.4°W to 50.6°S 35.1°W. Samples were collected in accordance with a 5-year permit for science operations and sampling (No. S3–3/2005) issued to the British Antarctic Survey by the British Foreign and Commonwealth Office under Article VII of the Antarctic Treaty and Article 17 of the Protocol on Environmental Protection to the Antarctic Treaty.

The methods for animal collection, the perturbation experiments, and pteropod shell analysis have been described in Bednarsek et al. [24], [26]–[27] and are only briefly summarized here. Juvenile and adult pteropods were collected with a variety of nets including a vertically hauled Bongo net with 100 and 200 µm meshed nets, a MOCNESS with 330 µm nets, and a towed Bongo with 300 µm and 600 µm meshed nets. Ship speed was between 1 and 2 knots during towing operations. The towed nets were generally more effective at capturing adult specimens, while juveniles were caught in both the vertical and towed nets. All deployments were carried out between 0 and 400 m. Captured pteropods were counted and examined under a light microscope to look for any instances of damage to the shell. Pteropods with evidence of cracks, pits, etchings, or perforations were excluded from the incubations.

### Control populations

Shelled pteropods are prone to mechanical damage, the two main causes being: (1) net sampling, and (2) incubation in the experimental chambers. This was accounted for by including two types of control in the experimental design: (1) natural environment control samples, caught in the upper 125 m, where Ω_ar_ = 1.82±0.60, made up from individuals without any sign of net-induced damage that were preserved in 70% ethanol immediately upon collection; (2) experimental control samples, which were individuals incubated in the same experimental setup as those in perturbed conditions but in which pCO_2_ (partial pressure of CO_2_) was maintained at present day levels. This was achieved through bubbling air with a 375 ppm (µmol/mol) CO_2_ mixing ratio through filtered seawater such that Ω_ar_ was 1.70±0.08. Undamaged pteropods were incubated in these conditions for eight days.

### Perturbation experiments

The effects of dissolution on shells of pteropods was determined at three different Ω_ar_ saturation states: (1) supersaturation, simulating present day Ω_ar_ levels; (2) transitional state, where Ω_ar_ was close to 1; and (3) undersaturation, where Ω_ar_ was less than 1. The incubation conditions were achieved by bubbling synthetic air containing CO_2_ mixing ratios (BOC Special Products) of 375, 500, 750, and 1200 ppm through filtered seawater from the ship's surface seawater supply that was previously filtered using a 0.22 µm GF/F filter. The bubbling was conducted either in 15 L carboys (for incubation of the adults) or in 2 L flasks (for incubation of the juveniles) and was carried out on deck at 3–4°C with the gas mixtures being introduced via micro-porous air-stones. The pCO_2_ in the headspace was measured using a LI-COR CO_2_/H_2_O analyser 6262. The bubbling was stopped and the porous stone apparatus was removed once the water had reached the target pCO_2_ before adding the pteropods. The flask was then sealed, taking care to reduce the headspace to a minimum. The flasks were kept at a constant temperature of 4°C and blacked-out for the duration of the experiments.

To ascertain the exact chemical perturbations achieved by the bubbling procedure, water was taken at the start and end of each experiment (250 ml+50 µl HgCl_2_ solution). These samples were analysed for total alkalinity (TA) and total dissolved inorganic carbon (DIC) using a VINDTA model 3C, calibrated with Certified Reference Materials (batch 76 from Andrew Dickson, Scripps Institute of Oceanography). The analytical precision and accuracy for TA was ±2 µmol kg^−1^ and ±4 µmol kg^−1^ respectively and, for DIC, ±1 µmol kg^−1^ and ±2 µmol kg^−1^ respectively. The remaining carbonate parameters were determined through application of the CO_2_SYS software (http://cdiac.ornl.gov/ftp/cp2sys) [28] using equilibrium constants from Mehrbach et al. [29] (as refitted by Dickson and Millero [30]) and the total pH scale. The error in pH_T_ was ±0.0062 and, in pCO_2_, ±5.7 µatm [31]. The start and end values of TA and DIC were used to determine an average Ω_ar_ value (± min/max value) for each perturbation experiment (Table 1).

**Table 1:** Physical and chemical parameters for the experiments; the values indicate the average and range (±) of two analyses, one of which was determined prior to the experiment and one after the experiment.

Treatment	Tempe-rature *(°C)*	Salinity	Phosphate *(µmol kg^−1^)*	Silicate *(µmol kg^−1^)*	TA *(µmol kg^−1^)*	DIC *(µmol kg^−1^)*	pH_T_	pCO_2_ *(µatm)*	HCO_3_^−^ *(µmol kg^−1^)*	CO_3_^2−^ *(µmol kg^−1^)*	Ω_ar_
**Natural control, live juveniles**	2.9±2.8	33.86±0.13	1.33±0.61	12.47±31.70	2290.6±3.7	2123.0±61.9	8.13±0.1	318±111	1983.1±91.9	121.6±39.6	1.82±0.6
**Exp. control (8 d) live juveniles**	4.0±2.0	33.83±0.02	1.55±0.25	10.41±2.11	2360.3±3.4	2211.5±12.7	8.07±0.0	387±24	2077.8±16.6	112.7±5.4	1.70±0.1
**Supersaturation (14 d) live juveniles**	4.0±2.0	33.83±0.02	1.55±0.25	10.41±2.11	2345.0±16.3	2220.0±1.4	8.00±0.1	455±50	2097.1±8.9	98.4±10.1	1.49±0.1
**Transitional state (14 d) live juveniles**	4.0±2.0	33.83±0.02	1.55+0.25	10.41±2.11	2353.2±15.8	2285.3±6.7	7.83±0.1	704±117	2177.5±11.3	69.7±10.9	1.06±0.2
**Transitional state (14 d) live adults**	4.0±2.0	33.83±0.02	1.55+0.35	10.41±.27	2298.0±30.0	2239.2±45.8	7.81±0.1	727±105	2135.4±46.8	64.2±6.5	0.97±0.1
**Undersaturation (4 d) live juveniles**	4.0±2.0	33.83±0.02	1.55+0.35	10.41±.27	2323.1±8.9	2295.5±5.4	7.70±0.0	940±27	2192.7±5.0	51.6±1.8	0.78±0.0
**Undersaturation (14 d) live juveniles**	4.0±2.0	33.83±0.02	1.55+0.35	10.41±.27	2330.3±6.4	2298.3±0.0	7.73±0.0	883±12	2191.0±4.4	54.8±0.9	0.83±0.0
**Undersaturation (14 d) dead juveniles**	4.0±2.0	33.83±0.02	1.55+0.35	10.41±.27	2317.9±11.9	2283.45±18.0	7.72±0.0	894±49	2181.3±17.4	53.9±1.9	0.81±0.0

### Pteropod shell analysis

**Figure 1:**
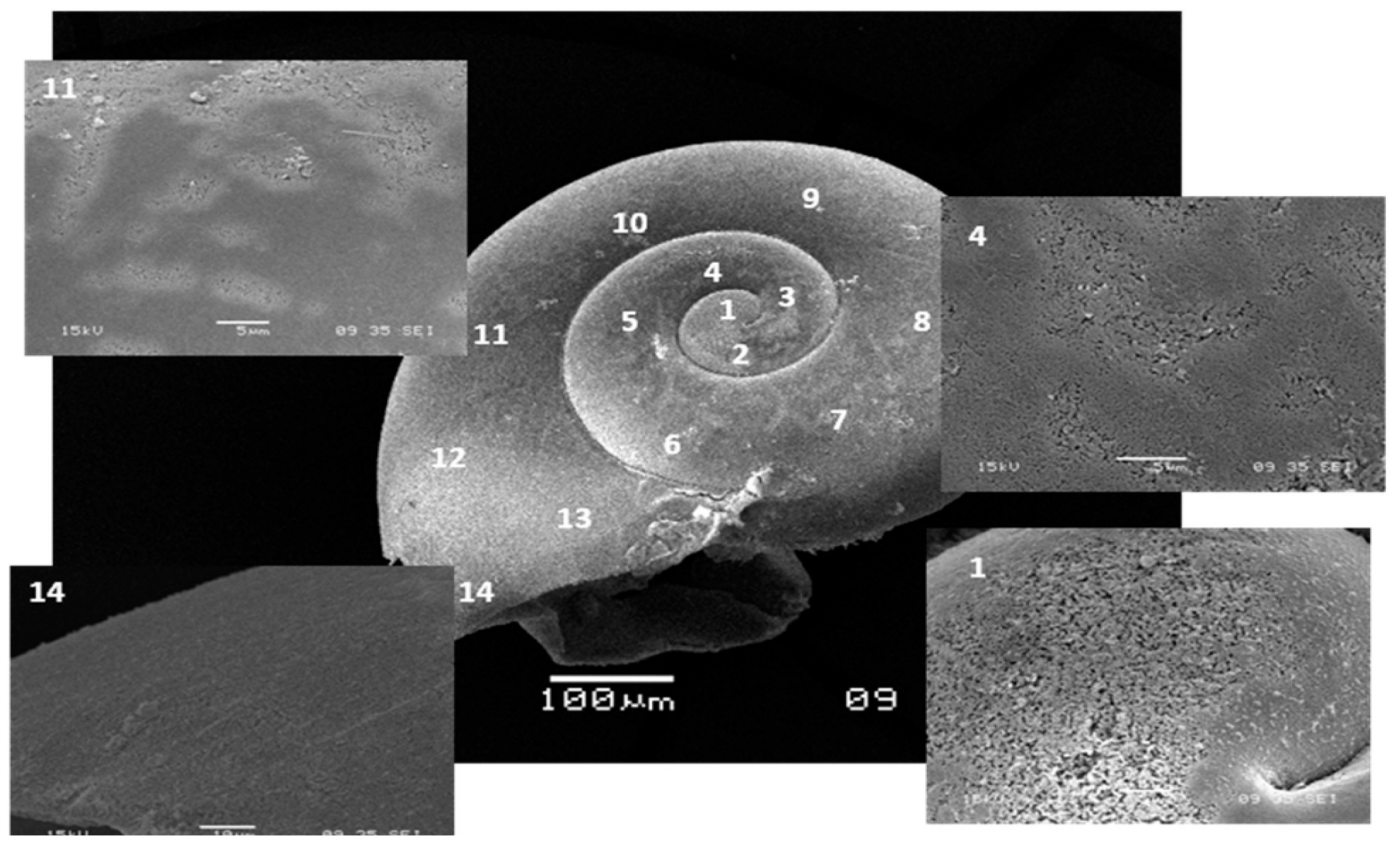
SEM micrograph of a *Limacina helicina antarctica* specimen with signs of surface shell dissolution. SEM micrograph exhibiting signs of dissolution at sites 1, 4, and 11 on the shell surface.

Pteropod samples were examined with scanning electron microscopy (SEM) to determine the level of shell dissolution, following Bednaršek et al. [26]. Abiogenic crystals were removed from the shells using 6% diluted hydrogen peroxide H_2_O_2_ followed by dehydration and drying using 2,2-dimethoxypropane and 1,1,1,3,3,3-hexamethyldisilazane. After the samples were mounted on an aluminium stub, oxygen plasma etching was carried out to expose microstructural elements of the shell and make them visible on the shell surface under SEM. This procedure was demonstrated to be non-destructive and very efficient. The SEM analysis was done with a JEOL JSM 5900LV fitted with a tungsten filament at an acceleration voltage of 15 kV and a working distance of about 10 mm [26]. The samples were gold coated in vacuo with a Polaron SC7640 sputter coater. Typical coating thicknesses ranged from 7 to 21 nm. The shell surface was examined by moving in small incremental steps around and across the shell and taking SEM micrographs (10–15 per animal). The first SEM micrograph was taken at the first whorl and the last at the growing edge (Fig. 1). A total of 38 animals were analysed equating to 60–90 micrographs per perturbation experiment. The SEM magnification was calibrated against known surface areas prior to any image analysis, upon which a calibration curve was produced [26]. This calibration curve was used to estimate the surface area of the shell.

### Dissolution of shell carbonate

To quantify the level of dissolution from SEM images, a number of further analytical steps were necessary, which we outline briefly. The calcified layers of the pteropod shell are made up of two layers: the outer prismatic layer and the inner crossed-lamellar layer. Previous work has shown that the level of dissolution of the prismatic and crossed-lamellar layers varied according to the type of dissolution observed (i.e., Type I, II, and III) [26]. We initially evaluated the extent of dissolution expressed as percentage of dissolved shell area of the three dissolution types, separately. This was then converted to the respective percentage of shell area over which the prismatic and crossed-lamellar layers were affected. The level of penetration into these layers was estimated through comparative measurements of size-normalized thickness in pristine and incubated shells. The percentage of shell lost to dissolution was expressed in terms of CaCO_3_ loss by applying previously reported relationships for length to shell weight and inorganic to organic carbon [27].

In pristine shells, the prismatic layer constituted 20% of the shell thickness and the crossed-lamellar layer, 80%. The level of dissolution of the calcified layers was only slight in Type I dissolution and those areas were not considered when calculating the level of CaCO_3_ lost to dissolution. For Type II dissolution, it was assumed that the prismatic layer (20% of shell mass) was completely dissolved but that the dissolution of the crossed-lamellar layer was only minor and constituted a negligible loss of CaCO_3_. In areas with Type III dissolution, it was implicit that the prismatic layer was completely dissolved, and that there was partial dissolution of the crossed-lamellar layer. The scoring of shells into areas of no dissolution, Type I, Type II or Type III dissolution was carried out “blind” (i.e., without knowledge of the treatment) and only related back to the treatment once the scores had been established.

**Figure 2:**
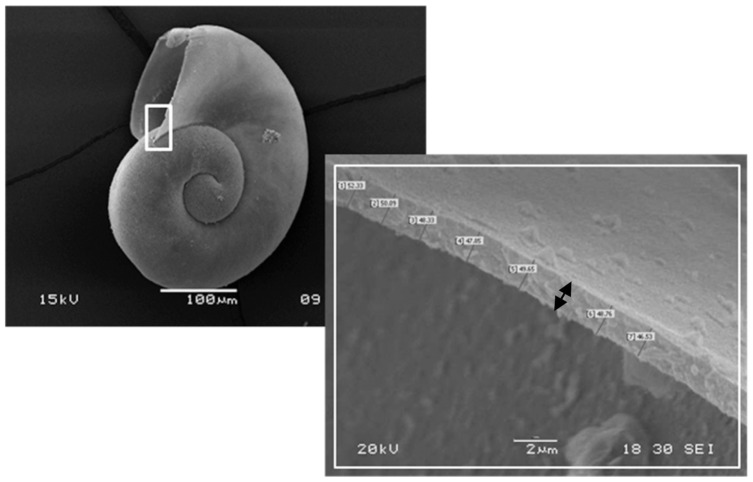
SEM micrograph of *Limacina helicina antarctica* showing the shell thickness measurements. SEM micrograph showing the shell thickness measurements with indication of the position where shell thickness measurements were made.

The next analytical stage was to determine the mean level of dissolution of the crossed lamellar layer in Type III dissolution. This was carried out at the shell aperture, where a natural cross-section of the shell could be resolved. All measurements were expressed as thickness-to-length (T/L) ratios to normalize for the shell size. Shell length measurements (expressed as shell diameter) were made at 90° to the direction of the shell aperture using a light microscope with a calibrated graticule (Fig. 2). Measurements of shell thickness were made at the same position of each shell aperture using SEM. Comparisons of T/L ratios were made between three sample sets: natural environment controls, experimental controls, and 14 day incubations of live juveniles held at undersaturation conditions. It is to be noted that this represented a subset of all experimental incubations given the time-intensive nature of these measurements. The proportional difference in T/L ratio values between the controls and the undersaturation incubation specimens was denoted as *u*. A mean value for *u* was determined across all measurements made on aperture cross-sections and was assumed to be the mean depth to which the prismatic plus the crossed-lamellar layer was dissolved in all areas exhibiting Type III dissolution (D*_TypeIII_*). Consequently, the proportion of shell dissolved as a result of Type II and Type III dissolution (D*_total_*) was calculated as follows:
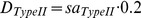
(3)

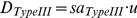
(4)

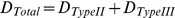
(5)where *D_TypeII_* and *D_TypeIII_* is the proportion of shell-loss resulting from Type II and Type III dissolution respectively, *sa* is the relative surface area of the shell affected by the different types of dissolution, and *u* is the relative thickness of the shell lost in areas of Type III dissolution and 0.2 represents the proportion of the shell mass within the prismatic layer. *D_Total_* represents the total proportion of shell lost per specimen. *D_Total_* actually represents minimum gross dissolution since this estimation method is also influenced by any ongoing calcification that would act to counter the gross amount of dissolution. As discussed later, this source of bias was likely to be minimal over the course of the incubations.

The next step was to convert proportional shell loss to absolute loss of shell mass. This required estimating initial shell mass (*M_0_*, mg) from measurements of shell length, achieved by applying the following conversion by Bednaršek et al. [27] for populations extracted from the study region:
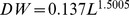
(6)where *DW* is dry weight in mg and *L* is shell diameter in mm.

*DW* was converted to total carbon mass by multiplying by a factor of 0.25 [32] and then partitioned into particulate inorganic carbon (PIC) and particulate organic carbon (POC) fractions by applying a ratio of 0.27∶0.73, as derived by Bednaršek et al. [27]. The mass of CaCO_3_ (i.e., *M_0_* in mg) was determined by multiplying PIC by 8.33, which is the molecular mass ratio of carbon to CaCO_3_. The daily mass loss of CaCO_3_ (*V*, mg d^−1^) from the shell during incubation was then calculated as:
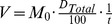
(7)where *t* is the duration of the incubation in days. The above term was alternatively expressed as the percentage of CaCO_3_ mass loss d^−1^ (*v*), which is *V·100* divided by *M_0_*.

### Changes to mineral structure

To determine whether there were any changes in the mineral structure at the growing edge of juvenile pteropod shells, Raman analysis was used to distinguish the occurrence of CaCO_3_ minerals other than aragonite. The samples were studied directly with no sample preparation. Instead, the growing edges of 25–30 animals from various experimental setups were examined in small incremental steps. The Raman bands for CaCO_3_ occur at ∼1085 Δcm^−1^ and a minor mode band at ∼155 Δcm^−1^. Aragonite has two additional bands at ∼207 and 704 Δcm^−1^. Laboratory measurements were performed with a laser Raman spectrometer, manufactured by Kaiser Optical Systems, Inc. The spectrometer and laser were connected to an optical probe head which was integrated into a Leica microscope with a 10 times magnifying objective (f/2.0. at 5.8 mm working distance, ∼50 µm spot size). Measurements were made on specimens exposed to manipulated conditions with high pCO_2_ levels to compare to those that were only exposed to ambient conditions.

### Statistical treatment

The data generated by the image segmentation analysis of shell surface dissolution were non-normally distributed and therefore were subjected to a square root transformation followed by an arcsine transformation [33]. Data on shell length and thickness were already converted to a ratio and so were not subjected to any further transformations. Datasets were analysed with t-tests provided they passed tests for normality (Kolmogorov-Smirnov test) and equal variance (variability about the group means). Non-normal datasets were otherwise analysed non-parametrically using a Mann-Whitney rank-sum test.

### Dissolution and calcification simulations

Our estimates of CaCO_3_ mass loss (*V*) under different aragonite saturation levels were compared to the findings of Comeau et al. [22], who estimated the rate of gross calcification in *L. helicina* exposed to varying conditions of aragonite saturation state and temperature. Comeau et al. [22] found a logarithmic relationship between aragonite saturation state and the amount of CaCO_3_ (*Q*, µmol (g wet weight)^−1^ h^−1^) precipitated, as follows:
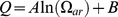
(8)where A is 0.57±0.4 and B is 0.25±0.02. We converted the calcification rate *Q* from Eq (8) for a range of Ω_ar_ values into mg CaCO_3_ per ind d^−1^ by firstly assuming an average shell diameter (*L*) of 0.31 mm [27] and determining the equivalent *DW* (mg) using Eq. (6). Wet weight (*WW*) was estimated by dividing *DW* by 0.28, following Davis and Wiebe [34]. The average *WW* of an individual was entered in Eq. (8) to derive the rate in terms of µmol CaCO_3_ ind^−1^ h^−1^. This was converted to mg CaCO_3_ ind^−1^ d^−1^ by applying a molar mass of 1 mole per 100.09 g and multiplying by 24 hours per day. Although there are known genetic differences between the Arctic and Antarctic populations of *L. helicina* [35], we assumed these to have a negligible effect on calcification rates in the present analysis.

Trajectories of mean shell weight were derived for two scenarios: first, for supersaturation levels (Ω_ar_ = 1.8), where only calcification would be performed; and second, for an undersaturation level of 0.8, where there would be both calcification and dissolution occurring. This level of undersaturation corresponds to the mean level achieved in the undersaturation incubations performed in the present study. It further represents the level of undersaturation that would prevail in the Southern Ocean surface waters by 2050 [14]. A period of 100 days for the trajectories was set based on the average productivity period at mid-latitudes in the Southern Ocean [36], when the majority of growth and development occurs in the pteropod population [27].

To estimate the rate of calcification using Eq. (8), it was necessary to estimate the growth in *WW* over the 100 day scenario. Hence, it was assumed that *WW* grew in direction proportion to shell weight, *M*, when in supersaturated conditions. For every daily increment in shell mass due to calcification, *m_calc_*, an increment of growth in *WW* was also added at a ratio of 1∶6.35, (*m_calc_*:*WW*). The next daily increment of *m_calc_* was then determined from the new *WW* and the process repeated, as follows:
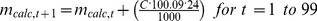
(9)where
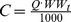
(10)and



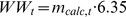
(11)


For the undersaturation scenario, it was assumed that growth in the non-shell fraction of *WW* remained the same but that growth in shell mass progressed at a different rate, as determined by Eq. (5).

The effect of gross dissolution (*m_diss_*) was determined as follows:
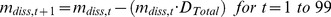
(12)


A trajectory of net shell mass (*M_t_*, mg CaCO_3_ ind^−1^) over the 100 day simulation period was determined as: 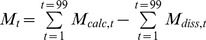
(13)


It is to be noted that *m_diss_* was assumed to be negligible in supersaturation conditions.

## Results

### Shell aperture analysis

**Figure 3:**
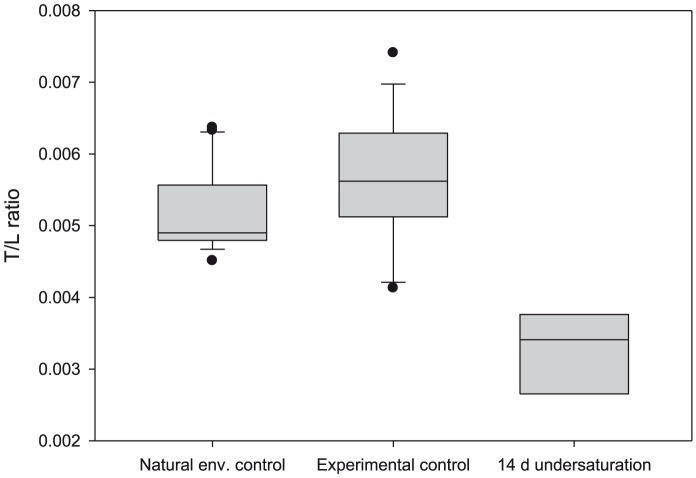
Thickness-to-length ratio of juvenile *Limacina helicina antarctica*. Thickness-to-length (T/L) ratio of juveniles from natural environment and experimental control populations, and from specimens incubated for 14 days in undersaturation conditions. Horizontal line represents the median; box limits, the 25^th^ and 75^th^ percentiles; whisker limits, 10^th^ and 90^th^ percentiles; and dots, the outliers.

We found that exposure to undersaturated conditions thinned the shell at the shell-aperture (Fig. 3). On average, specimens incubated for 14 days in undersaturated conditions were thinner by 39%±9% compared to specimens from the natural environment controls and the experimental controls. This value (expressed as a proportion) was assumed to be the value of *u* in all subsequent calculations of shell dissolution rates.

### Shell-surface analysis

#### Saturation state effects for juvenile pteropods

**Figure 4:**
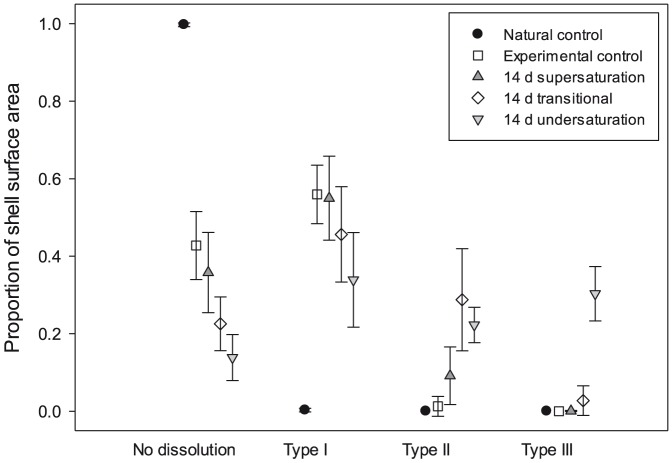
Proportion of shell surface dissolution in live juveniles incubated in different saturation conditions. Proportion of shell surface dissolution in live juveniles incubated in supersaturation conditions for 8 days and 14 days in different saturation conditions.

Levels of dissolution over the entire shell were quantified with image analysis. Pteropods in the experimental control displayed higher levels of Type I dissolution (56%±7 in the 8 day at Ω_ar_ = 1.70±0.1), compared to the natural control samples (0.3%, Fig. 4). This is likely to be a consequence of rearing pteropods in enclosed vessels where biological (respiration, calcification), chemical (dissolution) and physical (headspace-water CO_2_ exchange) processes altered the pCO_2_ level. Nevertheless, Type I dissolution represented only a minor loss of CaCO_3_ from the shell and was considered negligible in the development of dissolution regressions. Specimens kept at supersaturated conditions for 14 days (Ω_ar_ = 1.49±0.15) exhibited up to 55%±11% Type I and 9%±7% Type II dissolution, but there was no evidence of any Type III dissolution. Areas of Type III dissolution were present on specimens incubated at either Ω_ar_∼1 or in undersaturated conditions (Ω_ar_∼0.8), with more extensive dissolution in the latter.

#### Temporal effects

**Figure 5:**
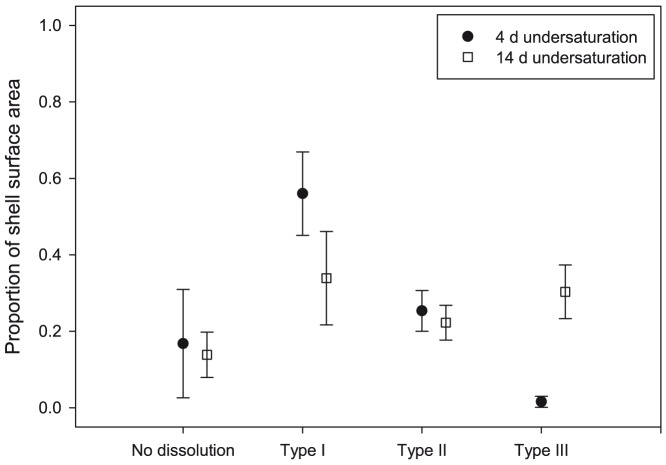
Proportion of shell surface dissolution in live juveniles incubated in undersaturation conditions for 4 and 14 days. Proportion of shell surface dissolution in live juveniles incubated in Ω_ar_ undersaturation conditions for 4 days or 14 days.

Type II and Type III dissolution was evident in juveniles incubated in undersaturated conditions for 4 days (Ω_ar_ = 0.78±0.03) and 14 days (Ω_ar_ = 0.83±0.02) (Fig. 5). The level of Type II dissolution was similar, at 27%±6% after 4 days and 25%±7% after 14 days. However, whereas only around 3%±3% of the shell was covered with Type III dissolution after 4 days, surface dissolution extended to 31%±6% after 14 days. Therefore, Type II dissolution occurred almost immediately on exposure to undersaturation conditions, whereas Type III dissolution mainly became apparent between 4 and 14 days.

#### Size and maturity effects

**Figure 6:**
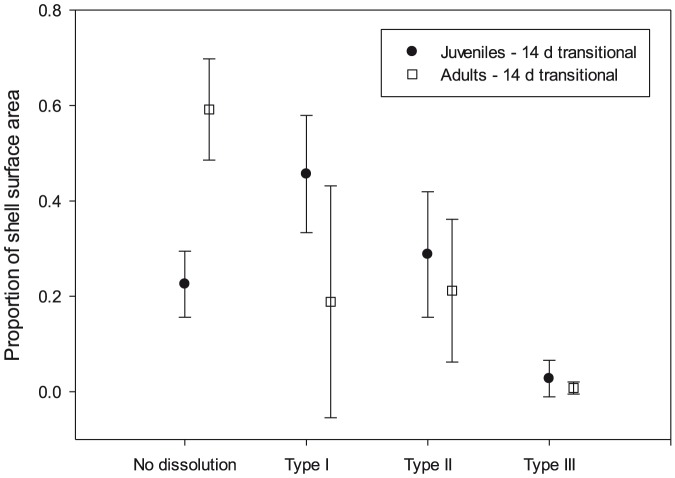
Proportion of shell surface dissolution in live pteropods incubated in transitional conditions for 14 days. Proportion of shell surface dissolution in live specimens that were either juvenile or adult, incubated in Ω_A_ transitional conditions for 14 days.

We found levels of Type I dissolution to be significantly greater in adults (59%±11%) than in juveniles (23%±7%) (Fig. 6; Normality and equal variance passed, t = −5.59, df = 5, P = 0.003). However, there was no significant difference in the levels of Type II and Type III dissolution between adults and juveniles (Type II: adult 21%±15% vs. juvenile 29%±13%, Type III: adult 1%±1% vs. juvenile 3%±4%; Normality and equal variance passed, t = −0.71, df = 5, P = 0.51 for Type II; t = 0.84, df = 5, P = 0.44 for Type III). Therefore, juveniles and adults showed the same Type II and Type III dissolution response at transitional saturation levels (Ω_ar_∼1).

### Changes in mineral structure

Aragonite was consistently observed in all the spectra made by Raman spectroscopy regardless of the Ω_ar_ saturation state. No mineral structure other than aragonite was found anywhere on the growing edge of any juvenile or adult pteropod. Therefore, the animals did not change their mineralization process in response to perturbations in the saturation state.

### Shell mass loss due to dissolution as a function of saturation state

The percentage shell mass loss due to dissolution over the course of the incubations was a minimum of 1.8%±3.1% under supersaturated conditions for 14 days to a maximum of 17.1%±3.0% in undersaturated conditions for 14 days (Table 2). In terms of shell mass loss d^−1^ (*v*), this equated to 0.1%±2% in supersaturated conditions, 0.5%±0.3% at Ω_ar_∼1 and between 1.2%±0.2% in undersaturated conditions (Ω_ar_∼0.8). The decrease in aragonite saturation levels from 1 to 0.8 therefore resulted in a two- to threefold increase in the rate of dissolution. When expressed in terms of equivalent loss in CaCO_3_ per individual, this is an increase from 0.07 µg d^−1^ to a maximum of 0.23 µg d^−1^ over the range of these saturation states.

**Table 2:** Calculation of the CaCO_3_ mass loss for various aragonite saturation states for G2 (Proportions were converted to percentages for clarity).

Pteropod length (mm)	0.320					
Pteropod weight (mg)	0.025					
Pteropod carbon (Larson, 1978)	0.006					
PIC (0.27 of carbon) (mg)	0.002					
POC (0.73 of carbon) (mg)	0.005					
CaCO_3_ mass (mg)	0.014					
**Dissolution in 4 days**	**Ω∼1.5**	**SD at Ω∼1.5**	**Ω∼1**	**SD at Ω∼1**	**Ω∼0.8**	**SD at Ω∼0.8**
Type II surface area (*_TypeII_*)					27.0%	(±6.0%)
Type III surface area (*_TypeII_*)					3.0%	(±3.0%)
Type II% shell loss (*D_TypeII_*)					5.4%	(±1.2%)
Type III% shell loss (*D_TypeIII_*)					1.2%	(±1.2%)
Total %shell loss (*D_Total_*)					6.6%	(±2.4%)
Total % shell loss d^−1^					1.6%	(±0.6%)
CaCO_3_ loss ind^−1^ d^−1^ (mg)					2.30E-04	(±8.31E-05)
**Dissolution in 14 days**	**Ω∼1.5**	**SD at Ω∼1.5**	**Ω∼1**	**SD at Ω∼1**	**Ω∼0.8**	**SD at Ω∼0.8**
Type II surface area (*sa_TypeII_*)	9.0%	(±6.0%)	29.0%	(±7.0%)	25.0%	(±13.0%)
Type III surface area (*sa_TypeII_*)	0.0%	(±3.0%)	3.0%	(±6.0%)	31.0%	(±1.0%)
Type II% shell loss (*D_TypeII_*)	1.8%	(±1.2%)	5.8%	(±1.4%)	5.0%	(±2.6%)
Type III% shell loss (*D_TypeIII_*)	0.0%	(±1.9%)	1.2%	(±2.3%)	12.1%	(±0.4%)
Total % shell loss (*D_Total_*)	1.8%	(±3.1%)	7.0%	(±3.7%)	17.1%	(±3.0%)
Total % shell loss d^−1^	0.1%	(±0.2%)	0.5%	(±0.3%)	1.2%	(±0.2%)
CaCO_3_ loss ind^−1^ d^−1^ (mg)	1.80E-05	(±2.37E-05)	6.97E-05	(±3.75E-05)	1.71E-04	(±2.99E-05)

This rate of % shell loss d^−1^ was most adequately represented by a 2-parameter exponential growth function, as follows: 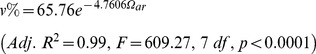
(14)


**Figure 7:**
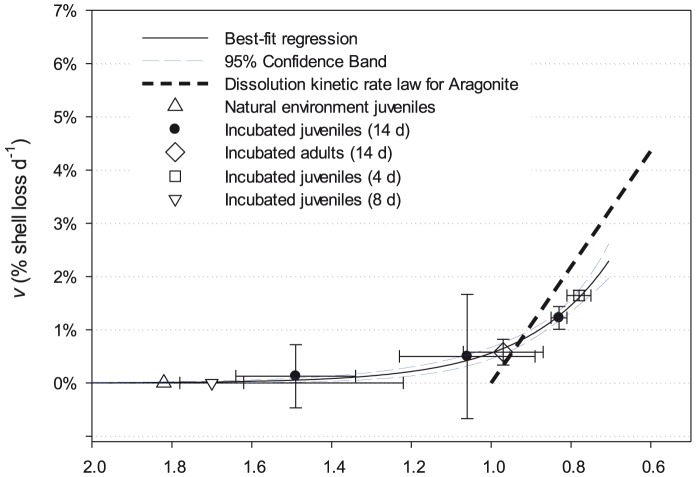
Percentage of shell mass loss across a range of aragonite saturation states. Shell mass loss d^−1^ (*v*) in live and dead specimens incubated between 4 and 14 days. Solid line represents a 2-parameter exponential function (±95% confidence intervals) fitted to all live specimens’ data points. The bold hashed line represents the dissolution kinetic rate law for aragonite. Error bars show ±1 SD on Ω_A_ values in the incubations and *v* respectively.

Both our observations and the fitted function show levels of dissolved shell loss at Ω_ar_ levels greater than 1, which was not predicted by the dissolution rate algorithm (Fig. 7). At Ω_ar_ levels below 0.9 however, the fitted function shows a slower increase in *v* shell loss d^−1^ than the rate kinetics. This reflects our observations that, for Ω_ar_ levels of around 0.8, *v* was between 0.7% and 1.7%, and not 2.2% as predicted by Eq. (2).

### Dissolution and calcification simulations

**Figure 8:**
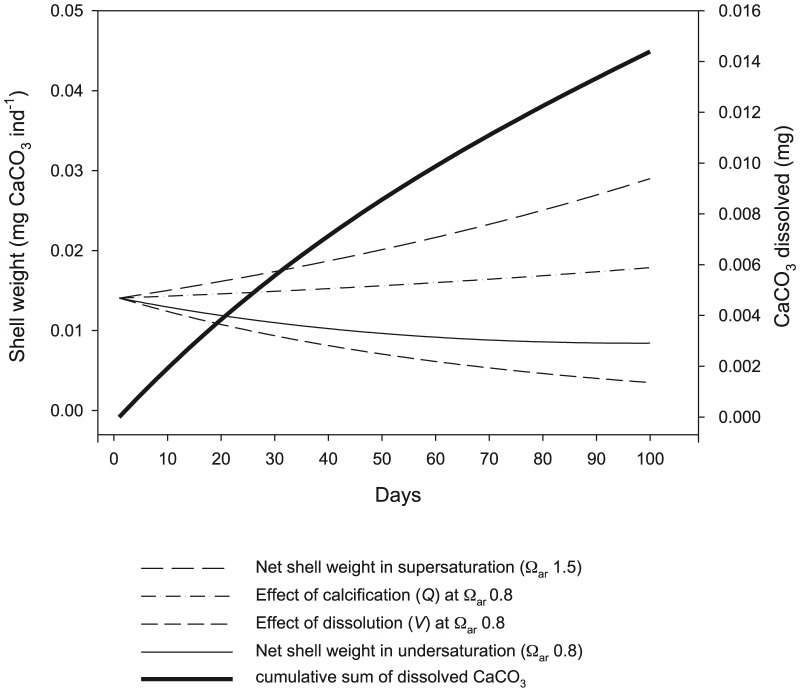
Simulation of dissolution and calcification on the shell weight exposed to supersaturation and undersaturation conditions. Simulation of the effect of dissolution and calcification on the shell weight when exposed to supersaturation or undersaturation conditions for 100 days. Bold line indicates the amount of CaCO_3_ dissolved over the course of the simulation where Ω_ar_ is 0.8.

To compare growth trajectories between saturation conditions, we simulated the effect of exposure to undersaturated (Ω_ar_∼0.8) compared to supersaturated conditions (Ω_ar_∼1.8) for 100 days (Fig. 8). It was assumed that any dissolution would be negligible when in supersaturation conditions, and shells grew through calcification according to the rate derived by Comeau et al. ([37]; Eq. (8)). *v* was set at 1.4% d^−1^, representing the average of dissolution observed on live specimens incubated for either 4 or 14 days at Ω_ar_∼0.8 (Table 2).

We found that the rate of gross calcification within undersaturated conditions compensated little for the loss of shell mass in these conditions. The total amount of CaCO_3_ lost by this juvenile pteropod through dissolution over 100 days would have been 0.014 mg.

### Weight-specific function for net calcification

**Figure 9:**
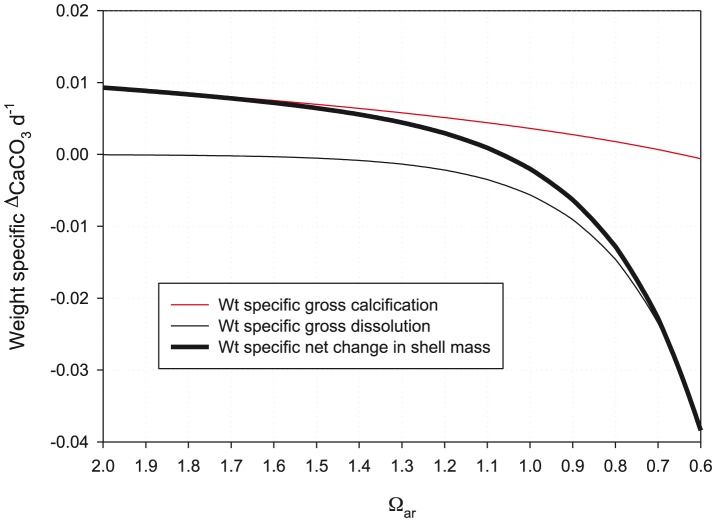
Weight-specific rates of net change processes as a function of aragonite saturation state. Weight-specific rates (d^−1^) of net change in shell mass as a function of aragonite saturation state.

The combined effects of gross dissolution and gross calcification on shell growth was examined through deriving trajectories for weight-specific net calcification against Ω_ar_ for *L. helicina* (Fig. 9). The trajectory was fitted closely by a three parameter exponential function as follows:
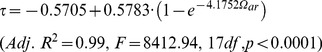
(15)where τ is weight-specific net calcification (d^−1^).

Whereas gross calcification ceases at an Ω_ar_ level of around 0.6, the additional influence of gross dissolution means that net calcification (*τ*) will become 0 at an Ω_ar_ level of approximately 1.

### Calculation of potential sinking flux

Declines in shell weight may in turn influence the level sinking flux. We estimated this through firstly assuming a standing stock of *L. helicina ant* of 32 mg C m^−2^, containing 9 mg m^−2^ of PIC, following Bednaršek et al. [24]. Applying a PIC:CaCO_3_ mass ratio of 8∶33 gives a mean population shell mass of 74.97 mg CaCO_3_ m^−2^. Bednaršek et al. [24], also derived a Production:Biomass ratio of 0.06 d^−1^, which, when applied to the population shell mass, gives a population shell mass production rate of 4.50 mg CaCO_3_ m^−2^ d^−1^. Based on observations made during this study, a juvenile exposed to Ω_ar_∼0.8 for 100 days would reduce in individual shell mass by 50%, which in turn would reduce the potential sinking flux to 2.25 mg CaCO_3_ m^−2^ d^−1^.

## Discussion

### Gross dissolution

In this study, we directly estimated the amount of CaCO_3_ shell lost to dissolution and found Type I dissolution was common in all incubations, although its absence from the natural control specimens indicated that at least some of this dissolution was an experimental artefact. Quantitatively, Type I dissolution represents a very minor loss of CaCO_3_ from the shell and can be ignored in terms of gross dissolution, while Type II and III dissolution represent a much greater amount of CaCO_3_ loss. The amount of shell surface covered by the latter two dissolution types increased with decreasing Ω_ar_ levels and longer periods of exposure. Nevertheless, when converted into a rate of shell mass loss due to dissolution, all undersaturated incubations (Ω_ar_∼0.8) resulted in a loss-rate of around 0.2 µg CaCO_3_ ind^–1^ d^−1^, which equates to approximately 1.4% of total shell mass d^−1^ regardless of the duration of the incubation.

### Shell loss mitigation processes

We found the dissolution response to Ω_ar_ undersaturated conditions to be relatively rapid; however, it was lower than predicted for the dissolution rate of abiogenic aragonite [6]. Like many molluscs, pteropods maintain an outer organic layer [38]–[39] that is analogous to the periostracum in molluscan groups such as the bivalves. In bivalves, the periostracum is comprised of chemically robust proteins and is believed to protect the shell from dissolution [40]. Partial chemical resistance and mechanical degradation could also be rendered through the multiple shell layers that provide both elasticity and hardness [39]. In addition, a microstructure with a higher organic content provides higher dissolution resistance through the shrouding of the crystals [41]. The full function of the outer organic layer in pteropods remains to be revealed but it appears to be able to offer some protection to the shell when faced with undersaturated conditions.

One possible means of dissolution mitigation is through ‘repair calcification’ [21], [25] where pteropods with shell dissolution were found to have affected areas repaired with new crystals. Although we found no evidence of repair, our methods were not ideal for resolving it. Furthermore, dissolution damage could be repaired over longer timescales on return to saturation conditions [21]. It is to be noted that, if repair calcification was taking place during the present incubations, this would result in an underestimation of the true value of gross dissolution since we assumed that specimens did not add any further shell mass over the course of the incubation.

### Functional response to undersaturated conditions

There have been a number investigations focused on the dissolution process, particularly targeting abiogenic aragonite (e.g., [6], [23], [42]–[46]). A study by Gangstø et al. [23] proposed an aragonite dissolution rate constant of 10.9 d^−1^. We compared the predictions of this rate to our own fitted relationship and found that abiogenic dissolution rate law only applies to Ω_ar_≤1. However, we also found evidence of dissolution at transitional saturation levels; in live juveniles, daily shell mass loss amounted to 0.5% in specimens incubated in Ω_ar_ = 1.03. The fitted function in turn reflected this transitional level mass loss, with the upward inflection starting at around Ω_ar_ of 1.3, reaching 0.6% d^−1^ at Ω_ar_ equal to 1. Such dissolution at transitional saturation levels has previously been reported by Betzer et al. [5] in the North Pacific, who found marked reductions in aragonite fluxes between two supersaturated depth horizons (100 m and 400 m), implying a loss in pteropod shell mass through shell dissolution. Although Betzer et al. [5] had no direct explanation for the pattern, they did refer to the findings of Morse et al. [45] and McGowan and Hayward [47], who proposed that freshly calcified aragonite surfaces in young pteropods (1–3 days old) are significantly more soluble than aged aragonite surfaces (30–70 days old). Greater solubility may therefore be expected in younger specimens or where new shell growth is occurring in older specimens.

There is a reasonable agreement to the predictions of the abiogenic rate of dissolution and our fitted function at Ω_ar_ levels just below 1. However, at increasing levels of undersaturation, we found that the abiogenic rate of dissolution overestimated the rate of shell mass loss in live organisms compared to observations. For instance, at Ω_ar_ of 0.8, juveniles showed a shell mass loss rate of between 0.8% and 1.5% d^−1^, whereas the rate law predicted 2.2% d^−1^. We advocate that it is unsafe to apply abiogenic dissolution rates when predicting the dissolution of aragonite in live organisms in biogeochemical models without also taking into account biological protection mechanisms.

### Net calcification

In incubations of *L. helicina* carried out in the Arctic, Comeau et al. [22] found that calcification continued even in undersaturated conditions down to Ω_ar_∼0.6. We demonstrated that the loss from dissolution would be twice the amount contributed by net calcification, leading to a net decrease in the mass of the shell of 0.007 mg (50% of original shell mass) over the 100-day simulation period.

Comeau et al. ([48], their Table 3) made projections under the IPCC (Intergovernmental Panel on Climate Change) SRES (Special Report on Emission Scenarios) A2 scenario for anthropogenic CO_2_ emissions on the rates of gross calcification by pteropods at a number of oceanic sites where *L. helicina* has been caught, including sites in the Arctic and Antarctic. At one site in the Arctic (83.58° N, 98.58° W), the projection was for Ω_ar_ to drop to 0.4 by 2095, by which point gross calcification in pteropods would cease. At another Arctic site (Svalbard, 79.8° N, 11.8° E) and in the Southern Ocean (62.8° S, 60.8° E), the prognosis for 2095 was for Ω_ar_ to drop to 1.1 and for gross calcification to continue at a rate of between 50 and 60% of the preindustrial rate. According to the net calcification function derived by the present study, such Ω_ar_ levels would result in *L. helicina* being incapable of calcifying enough to offset dissolution. They would be unable to grow in shell mass in any of these polar oceanic regions that they typically inhabit.

### Influence of net calcification on net aragonite flux

We estimated potential sinking fluxes of 4.50 mg CaCO_3_ m^−2^ d^−1^ in supersaturated conditions (Ω_ar_∼1), and 2.25 mg CaCO_3_ m^−2^ d^−1^ in undersaturated conditions (Ω_ar_∼0.8), assuming that juveniles are exposed to undersaturated conditions over a 100 day productivity period. Attempts at measuring pteropod sinking flux have been made by determining accumulation rates of bottom sediments [49]–[50] or vertical fluxes measured with sediment traps [5], [49] but these approaches have been criticised because of the combined effects of dissolution in deeper layers and predation [5], [51]–[54]. As an alternative, sinking fluxes can be determined based on productivity rates or instantaneous growth rates [55]–[56]. At Ocean Station PAPA, aragonite production was measured at 4.4 mg CaCO_3_ m^−2^ d^−1^, split between *L. helicina* (2.6±0.3) and *Clio pyramidata* (1.8±0.2) [55]. Similar levels were found in the Bahamas (2.8±0.3 mg CaCO_3_ m^−2^ d^−1^), the equatorial Pacific (6.6±1.2 mg CaCO_3_ m^−2^ d^−1^), and the Central Pacific (1.4±0.6 mg CaCO_3_ m^−2^ d^−1^) [56]. On average, productivity values were around 0.5 mg CaCO_3_ m^−2^ d^−1^ greater than estimates made from sediment traps in the same regions [5], [56]. 50% decrease in the sinking flux that we predict would occur under undersaturated conditions would have a much greater significance to the overall carbonate cycle in the Southern Ocean, as well as other high-latitude regions, where pteropods are found in high abundances.

Accompanying the decrease in overall shell mass is also the decrease in shell weight in terms of how fast it will sink through the water column. Byrne et al. [6] proposed that the decrease in sinking rate scales with loss of mass, as follows:
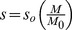
(16)where *s* is the revised sinking velocity (cm s^−1^), *s_o_*, the original sinking velocity (cm s^−1^), *M*, the remaining shell mass (mg CaCO_3_), and *M_0_*, the original shell mass (mg CaCO_3_).

Byrne et al. [6] measured the sinking speed of *Limacina inflata*, of the same size as juvenile *L. helicina ant.* in the present study (∼0.3 mm shell diameter, ∼0.014 mg CaCO_3_) to be 1.4 cm s^−1^. Exposure to Ω_ar_ levels of 0.8 for 100 days would reduce shell mass to 0.007 mg CaCO_3_, resulting in the sinking speed being reduced to 0.7 cm s^−1^. As a consequence, the partially dissolved shell would take twice the amount of time to sink to the bottom of a 3000 m water column (5.7 days versus 2.5 days). In undersaturated conditions, the level of dissolution in these lighter shells will be even greater, making their sinking rates even slower. With respect to the carbonate cycle, slower sinking speed will result in a longer retention time in the upper water column, which may have a mitigating effect in neutralising anthropogenically induced acidification of mid-water depths [8]–[9], [49], [57]. Nevertheless, the lighter, slower-sinking pteropods would have a diminished impact in their role as ballast to sinking particulate organic matter [16]. This will result in greater subsurface water column remineralization of this particulate organic material and, ultimately, a less effective carbon pump.

### Concluding remarks

In modelling the sensitivity of pelagic calcification to ocean acidification, Gangstø et al. [23], [58] determined that anthropogenic CO_2_ emissions may lead to irreversible changes in Ω_ar_ for several centuries. Even under optimistic emission scenarios, the ratio of open-water CaCO_3_ dissolution will continue until 2500 where it will be 30–50% higher than at pre-industrial times. The consequence is a severe loss of suitable habitat for aragonite calcifiers. This in turn will result in a depletion of the rate of carbon and carbonate flux to the deep ocean. As confidence intervals of future projections increasingly narrow, the argument is progressing beyond whether suitable habitat will be lost to when and to what extent. The application of results obtained in this study will now enable regions of imminent habitat loss to be identified and monitored and the consequences to the sinking flux to be estimated.
